# Membrane Potential Controls Adipogenic and Osteogenic Differentiation of Mesenchymal Stem Cells

**DOI:** 10.1371/journal.pone.0003737

**Published:** 2008-11-17

**Authors:** Sarah Sundelacruz, Michael Levin, David L. Kaplan

**Affiliations:** 1 Department of Biomedical Engineering, Tufts University, Medford, Massachusetts, United States of America; 2 Biology Department, and Tufts Center for Regenerative and Developmental Biology, Tufts University, Medford, Massachusetts, United States of America; University of Michigan, United States of America

## Abstract

**Background:**

Control of stem cell behavior is a crucial aspect of developmental biology and regenerative medicine. While the functional role of electrophysiology in stem cell biology is poorly understood, it has become clear that endogenous ion flows represent a powerful set of signals by means of which cell proliferation, differentiation, and migration can be controlled in regeneration and embryonic morphogenesis.

**Methodology/Principal Findings:**

We examined the membrane potential (V_mem_) changes exhibited by human mesenchymal stem cells (hMSCs) undergoing adipogenic (AD) and osteogenic (OS) differentiation, and uncovered a characteristic hyperpolarization of differentiated cells versus undifferentiated cells. Reversal of the progressive polarization via pharmacological modulation of transmembrane potential revealed that depolarization of hMSCs prevents differentiation. In contrast, treatment with hyperpolarizing reagents upregulated osteogenic markers.

**Conclusions/Significance:**

Taken together, these data suggest that the endogenous hyperpolarization is a functional determinant of hMSC differentiation and is a tractable control point for modulating stem cell function.

## Introduction

Harnessing the potential of stem cells for applications such as wound healing and tissue regeneration is a tantalizing yet daunting task. During embryonic development and tissue regeneration, two events during which stem cells actively proliferate and differentiate, a wealth of literature suggests that biophysical signaling plays a critical role (reviewed in [1], [2]). For example, during both limb bud development in amphibians and mammals and spontaneous limb regeneration in adult urodeles, limbs establish highly localized endogenous electric fields, which, if disrupted by an exogenous current, results in deformed structures [3]–[9]. There is evidence that application of exogenous electric fields can augment regeneration or even induce a degree of regeneration in normally non-regenerative species [10]–[12].

Recent application of molecular genetics to this field has confirmed the crucial role of bioelectric signals in regenerative processes, identified the ion transporters responsible for generating instructive ion flows, and characterized downstream changes in gene expression. For example, in *Xenopus* tail regeneration [13], the vacuolar H^+^-ATPase pumps a strong H^+^ flux through the cell membrane of regeneration bud cells. This very early process controls downstream changes in cell proliferation, regeneration-specific gene expression, and axonal patterning, and is both necessary and sufficient for regeneration of spinal cord, muscle, and vasculature in the tail.

Underlying the complex processes of tissue development and regeneration are individual cellular events such as proliferation, migration, and differentiation, which themselves may be regulated by biophysical signaling. For example, in a study of cell cycle regulation in fibroblasts, activity of the Na^+^-H^+^ exchanger NHE1 caused an increase in intracellular pH, which regulated the timing of the cell cycle G_2_/M transition and resulted in cell proliferation [14]. In a study of nerve growth cone migration, Rho GTPases mediated growth cone steering in electric fields, linking membrane receptor signaling pathways to spatial regulation of the cytoskeleton [15]–[18]. In a corneal wound healing model, endogenous electric fields regulated both cell migration [19] and the orientation and frequency of cell division [20]. Phosphoinositide 3-kinase (PI(3)K) and Src signaling pathways mediated this electrotactic response. Disruption of the gene for PI(3)Kã resulted in diminished electrotactic migration, while disruption of the gene PTEN (phosphatase and tensin homolog) resulted in enhanced migration. These are the first-known genes to control electric-field-directed cell migration. These studies and others have shown the importance of biophysical signaling and have uncovered the mechanisms by which biophysical signals are translated into familiar signaling pathways.

One exciting application of biophysical signaling is in the control of stem cell behavior. Studies have shown that stem cells exhibit unique electrophysiological profiles in their undifferentiated state [21]–[25]. More interestingly, ionic currents and channels have been found to play important roles during myoblast, cardiomyocyte, and neural stem cell differentiation [21], [25]–[28]. However, the ability of these endogenous electrical signals to act as a functional biophysical control mechanism in stem cell biology is poorly understood. Moreover, it is not known whether stem cells' differentiation process is controlled by the electric fields, localized pH and ion gradients, or transmembrane potential changes resulting from the activity of ion channels and pumps. The aim of this study was to characterize membrane potential (V_mem_) changes in human mesenchymal stem cells over the course of differentiation toward two different cell lineages, bone and fat, and to investigate a functional relationship between control of membrane potential and differentiation.

## Results

### hMSCs show different membrane potential profiles during OS vs. AD differentiation

**Figure 1:**
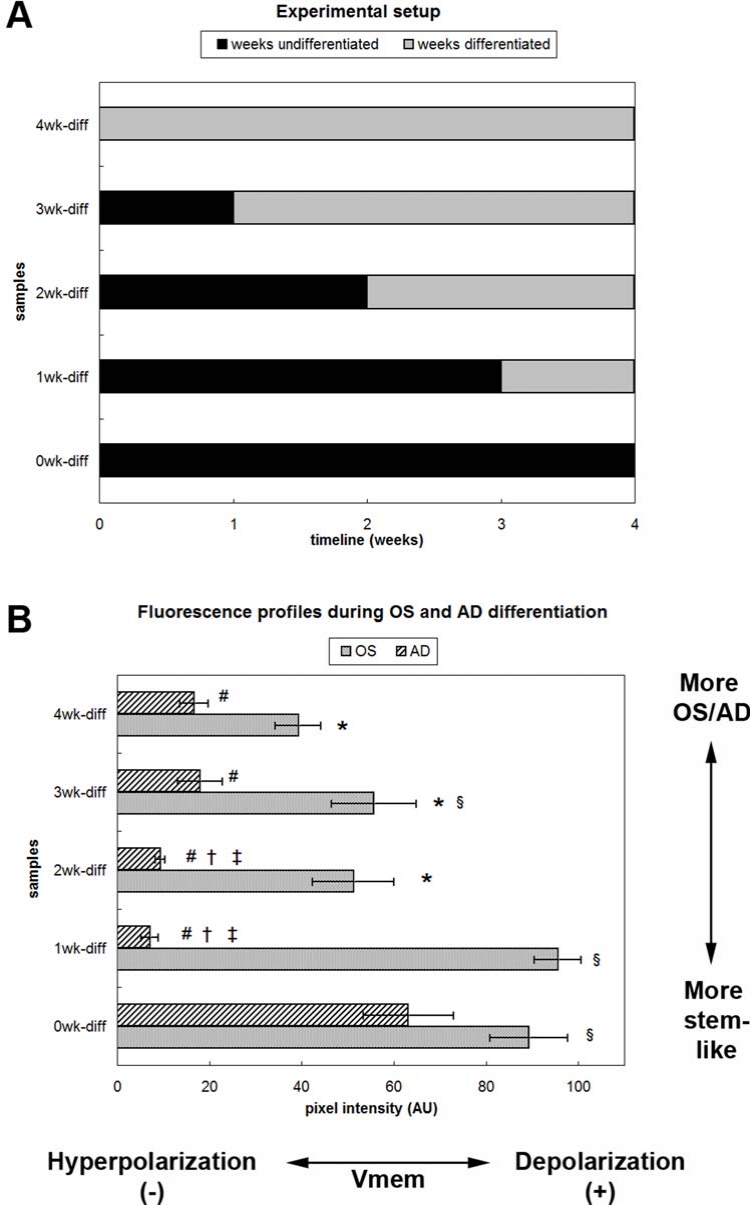
V_mem_ hyperpolarization exhibited by OS- and AD-differentiated cells. (A) Cell culture timeline for V_mem_ studies. Cells were seeded in control medium, then switched to OS or AD differentiation medium (OS or AD) at various time points over the course of 4 weeks. After 4 weeks, cells that had differentiated for a total of 0, 1, 2, 3, or 4 weeks (samples 0wk-diff, 1wk-diff, 2wk-diff, 3wk-diff, and 4wk-diff, respectively) were imaged on the same day. (B) Fluorescence measurements from cells cultured according to the timeline in OS or AD media. Cells were stained with the voltage-sensitive dye DiSBAC, which exhibits higher intensity with membrane depolarization. Data points are mean pixel intensity±standard deviation (N = 5–15 cell fields). Marked samples are statistically different, * relative to 0wk-diff OS sample (p<0.0005), § relative to 4wk-diff OS sample (p<0.0005), # relative to 0wk-diff AD sample (p<0.0005), † relative to 3wk-diff AD sample (p<0.0002), ‡ relative to 4wk-diff AD sample (p<0.005).

**Figure 2:**
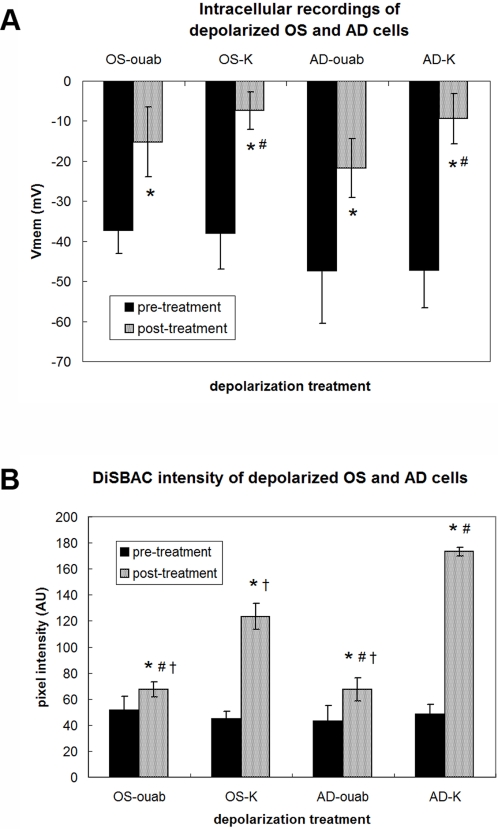
Measurement of resting and depolarized membrane potentials during OS and AD differentiation. (A) Intracellular recordings of resting and depolarized membrane potentials (V_mem_) in hMSCs during OS and AD differentiation. Cells were impaled individually and the V_mem_ recorded until a stable baseline was reached (pre-treatment), then 10 nM ouabain (OS-ouab, AD-ouab samples) or 80 mM K^+^ (OS-K, AD-K) was added and the V_mem_ recorded until a new equilibrium was reached (post-treatment). Data points are mean potentials±standard deviation (N = 6–7 cells). Marked samples are statistically different, * relative to respective pre-treatment samples (p<0.03), # relative to AD-ouab post-treatment sample (p<0.04). (For clarity, statistical significances marked by # are reported among post-treatment samples only.) (B) Intensities of DiSBAC_2_(3)-loaded cells at resting and depolarized potentials during OS and AD differentiation. Pre-treatment values are the fluorescence intensities of OS and AD cells at rest, while post-treatment values are the fluorescence intensities after depolarization with 10 nM ouabain (OS-ouab, AD-ouab) or 80 mM K^+^ (OS-K, AD-K). Data points are mean pixel intensity±standard deviation (N = 15–20 cell fields). Marked samples are statistically different, * relative to respective pre-treatment samples (p≪0.0001), # relative to OS-K post-treatment sample (p≪0.0001), † relative to AD-K post-treatment sample (p≪0.0001). (For clarity, statistical significances marked by # and † are reported among post-treatment samples only).

In order to determine whether membrane potential of hMSCs changes as a function of differentiation time, we tracked membrane potential changes during osteogenic (OS) and adipogenic (AD) differentiation with confocal microscopy using the voltage-sensitive fluorescent dye DiSBAC_2_(3). Since DiSBAC_2_(3) is an anionic bis-oxonol, it tends to partition into the cell membrane when the intracellular charge is more positive, resulting in a higher fluorescence signal when the cell is depolarized. The fluorescence profile during OS differentiation showed decreased membrane potential (hyperpolarization) compared to undifferentiated cells after 2, 3, and 4 weeks of differentiation, with the 4-week-differentiated samples being the most hyperpolarized (Fig. 1). During AD differentiation, all differentiated AD cells were hyperpolarized compared to undifferentiated cells, but the extent of hyperpolarization varied with differentiation time. AD cells exhibited slightly greater hyperpolarization with 1–2 weeks of differentiation vs. with 3–4 weeks of differentiation. Due to technical difficulties, we were not able to obtain direct intracellular recordings of the differentiating cells beyond the first week of culture. However, we estimated V_mem_ changes from DiSBAC_2_(3) fluorescence changes by equating a 1% change in fluorescence to a 1 mV change, as has been shown for the closely-related voltage-sensitive dye DiBAC_4_(3) [29]. From these voltage changes, V_mem_ values at weeks 1, 2, 3, and 4 can be calculated with respect to V_mem_ values at week 0, which were successfully recorded from cells during Days 1–4 of OS or AD differentiation (Fig. 2A). From these estimates, OS and AD cells exhibited a net V_mem_ hyperpolarization of 56 and 74 mV, respectively, at the end of four weeks of differentiation (Table 1). From these data, we conclude that differentiated hMSCs are hyperpolarized compared to undifferentiated hMSCs, and that the polarization of the cells is associated with their differentiation toward different cell lineages (OS or AD).

**Table 1:** Estimated V_mem_ values during OS and AD differentiation.

time differentiated (wks)	OS Vmem (mV)	AD Vmem (mV)
0	−37.0±9.4	−47.0±15.5
1	−30.0±5.7	−135.8±2.9
2	−79.7±9.9	−132.2±1.7
3	−74.6±10.3	−118.5±7.6
4	−93.0±5.5	−120.7±4.8

V_mem_ values for week 0 were taken from intracellular recordings of cells during early differentiation (Days 1–4) (Fig. 2A). Subsequent V_mem_ values were estimated from the DiSBAC fluorescence data (Fig. 1B): since bis-oxonol dyes such as DiSBAC typically generate a ∼1% change in fluorescence per 1 mV change [29], changes in V_mem_ for weeks 1–4 were estimated by calculating the percentage changes in fluorescence compared to week 0.

### V_mem_ measurements in resting and depolarized cells during OS and AD differentiation

After observing trends in membrane potential changes during hMSC differentiation (a hyperpolarized phenotype in differentiated cells vs. undifferentiated cells), we sought to determine whether this hyperpolarization was functionally required for differentiation. To accomplish this, we disrupted the normal progression of membrane potential changes by depolarizing hMSCs during differentiation. Two independent strategies were employed to depolarize membrane potential to ensure that the observed effects were in fact due to membrane depolarization, rather than due to inducer-specific effects. Thus, hMSCs were cultured and differentiated in the presence of high [K^+^]_out_ or ouabain to achieve depolarization. Normally, the ratio between extracellular and intracellular [K^+^] is small (low [K^+^]_out_/[K^+^]_in_). However, as [K^+^]_out_ is elevated, the ratio increases as [K^+^]_out_ approaches [K^+^]_in_. As this ratio approaches 1, the magnitude of the Nernst potential for K^+^ decreases, effectively raising the membrane potential closer to 0 mV (depolarization). Increasing the extracellular potassium concentration is a standard means of depolarizing cells. Similarly, blockade of the the Na^+^/K^+^ ATPase pump by the specific inhibitor ouabain was used to inhibit the main source of transmembrane potential. Samples were collected after 2, 7, 14/15 and/or 22 days of differentiation for PCR and other tissue-specific analyses. Treated cells were compared to untreated differentiated cells (AD or OS) or to undifferentiated cells (control).

We first confirmed the activity of our depolarizing treatments using direct electrophysiology. Intracellular recordings were taken from MSCs cultured in OS or AD medium at early time points during differentiation (Days 1–4) (Fig. 2A). On average, OS cells were at a resting potential of −37 mV. Exposure to 10 nM ouabain depolarized the cells to −15 mV, while exposure to 80 mM K^+^ depolarized the cells to −7 mV. On average, AD cells were at a resting potential of −47 mV. Exposure to 10 nM ouabain depolarized the cells to −22 mV, while exposure to 80 mM K^+^ depolarized the cells to −9 mV. Confocal imaging of DiSBAC-loaded cells also reported depolarization (higher pixel intensity) for OS and AD cells exposed to ouabain and high K^+^ (Fig. 2B). We then tested the effects of these treatments on the differentiation trajectory of the hMSCs.

### Depolarization inhibits AD differentiation

The effect of depolarization on AD differentiation was assessed by quantifying the expression of the AD-related genes peroxisome proliferator activated receptor γ (PPARG) and lipoprotein lipase (LPL). PPARG is a an adipogenic-specific transcription factor in the nuclear hormone receptor superfamily [30], [31]. Upregulation of PPARG expression is induced during mitotic arrest very early in adipose differentiation and stimulates the adipogenic pathway, activating the transcription of other adipose-specific genes [32]–[34]. LPL is one of several highly specialized proteins induced during adipocyte differentiation by PPARG, and plays an important role in lipoprotein and energy metabolism [35], [36]. LPL hydrolyzes triglyceride moieties of lipoproteins, generating free fatty acids necessary for triglyceride synthesis and lipid accumulation [37].

### Depolarization by high extracellular [K^+^] suppresses AD differentiation

**Figure 3:**
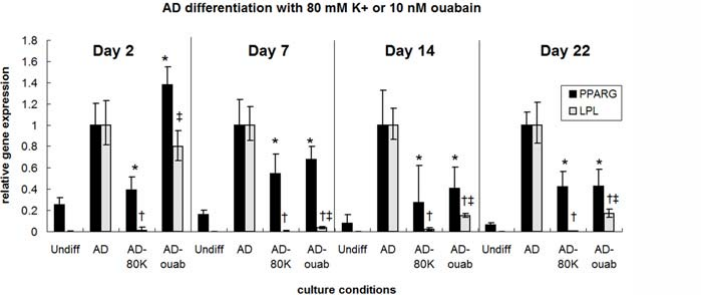
Depolarization suppresses AD gene expression. PPARG and LPL expression were suppressed on Days 2, 7, 14, and 22 by addition of 80 mM K^+^ (AD-80K) during AD differentiation. Similarly, PPARG and LPL expression were suppressed on Days 7, 14, and 22 by addition of 10 nM ouabain (AD-ouab) during AD differentiation. Data points are mean relative expression±standard deviation (N = 6). Marked samples are statistically different, * relative to PPARG expression of untreated AD samples (p<0.05), † relative to LPL expression of untreated AD samples (p<0.003), ‡ relative to LPL expression of AD-80K samples (p<0.0005). (For clarity, statistical significances are reported among samples taken within the same day.) Undiff, hMSCs cultured in control medium; AD, hMSCs cultured in AD medium; AD-80K, hMSCs cultured in AD medium supplemented with 80 mM K^+^; AD-ouab, hMSCs cultured in AD medium supplemented with 10 nM ouabain.

**Figure 4:**
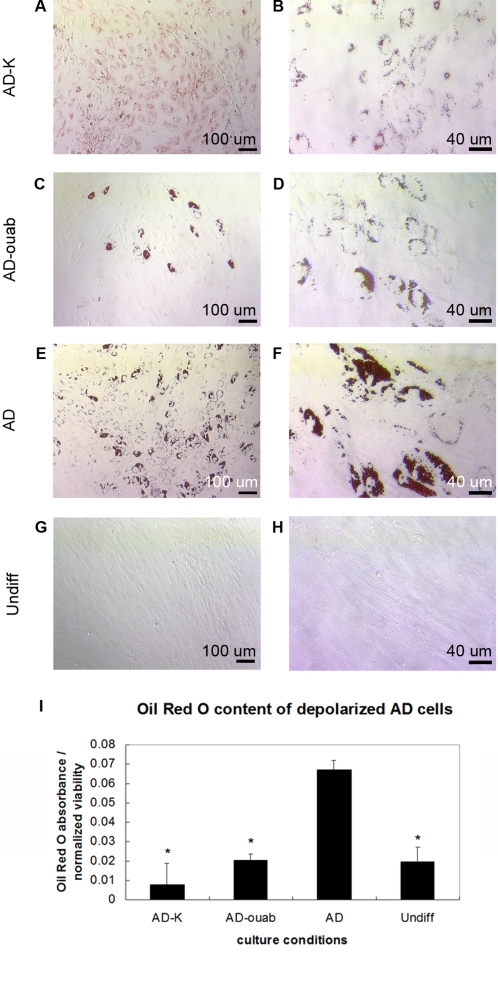
Depolarization reduces fat droplet accumulation during AD differentiation. Oil Red O staining revealed less accumulation of fat droplets in K^+^- and ouabain-treated AD cells. Depolarized cells were cultured in AD medium supplemented with 80 mM K^+^ (AD-K; images A, B) or 10 nM ouabain (AD-ouab; images C, D). Control cells were cultured in AD medium (AD; images E, F) or control medium (Undiff; images G, H). After 7 days, cells were stained with Oil Red O and imaged at magnifications of 100× (images A, C, E, G; scale bar = 100 µm) and 320× (images B, D, F, H; scale bar = 40 µm). Oil Red O was then extracted from each sample and measured spectrophotometrically (I). Data points are mean absorbance±standard deviation (N = 6). Marked samples are statistically different, * relative to untreated AD samples (p<0.009).

Potassium gluconate was added to AD culture medium to elevate the extracellular concentration of K^+^. On Days 2, 7, and 14, samples treated with 80 mM K^+^ (denoted AD-80K) had significantly lower LPL expression compared to untreated AD samples (decrease of 50.7-fold, 219.7-fold, and 43.5-fold, respectively, p<0.007, Fig. 3). Oil Red O staining on Day 7 also resulted in a different staining pattern in K^+^-treated AD cells: staining was diffuse and fairly uniform throughout the sample, revealing tiny lipid droplet formation mostly at the periphery of the cells (Fig. 4A,B), compared to the more extensive lipid droplet formation in AD cells (Fig. 4E,F). Oil Red O absorbance levels were also reduced 8.7-fold (p<0.002, Fig. 4I). On Day 22, LPL levels of depolarized samples continued to remain lower than untreated AD samples (83.9-fold, p≪0.001). These results demonstrate that exposure to 80 mM extracellular K^+^, which depolarizes hMSCs, can suppress AD differentiation for approximately three weeks.

### Depolarization by ouabain suppresses AD differentiation

To ensure that the observed effects were not due specifically to the high extracellular K^+^ levels but rather to the reduction of transmembrane potential, we next depolarized cells by a different mechanism. Since activity of the Na^+^/K^+^ ATPase pump is the main source of V_mem_ gradient in most cells, effects of the Na^+^/K^+^ ATPase inhibitor ouabain were assessed during AD differentiation. On Day 2, cells treated with 10 nM ouabain (denoted AD-ouab) had higher PPARG levels (1.9-fold, p<0.001) but similar LPL levels compared to untreated AD cells (Fig. 3). By Days 7, 14, and 22, AD-ouab cells had lower PPARG levels (1.5-, 2.5-, and 2.4-fold, respectively, p<0.05) and lower LPL levels (25.4-, 7.2-, and 6.2-fold, respectively, p<0.008) compared to untreated AD cells. On all days, however, LPL expression in AD-ouab cells, while lower than that of untreated AD cells, was greater than that of AD-80K cells (34.0-, 7.0-, 5.2-, and 13.5-fold greater for Days 2, 7, 14, and 22, respectively, p<0.0005, Fig. 3). Oil Red O absorbance on Day 7 also showed a 3.3-fold decrease in ouabain-treated AD cells compared to untreated AD cells (p<0.007, Fig. 4I). Unlike K^+^-treated cells, AD-ouab cells did not stain uniformly (Fig. 4C,D). Only a small population of cells stained for lipid droplets, but those that were positively stained showed more extensive droplet accumulation compared to K^+^-treated cells. The greater staining intensity of this subpopulation may explain why the Oil Red O quantification in AD-ouab cells was slightly higher than that of K^+^-treated cells, even though the K^+^-treated cells had a larger population of positively stained cells (Fig. 4I). Apart from this subpopulation, however, the majority of AD-ouab cells did not show any lipid droplet formation.

Therefore, treatment with 10 nM ouabain also suppresses AD differentiation, although not to the same degree as treatment with 80 mM K^+^. Some cells were partially able to overcome the effects of ouabain depolarization at later time points, as seen by their ability to increase LPL expression and form lipid droplets.

### Shorter durations of depolarization are sufficient to suppress AD differentiation

**Figure 5:**
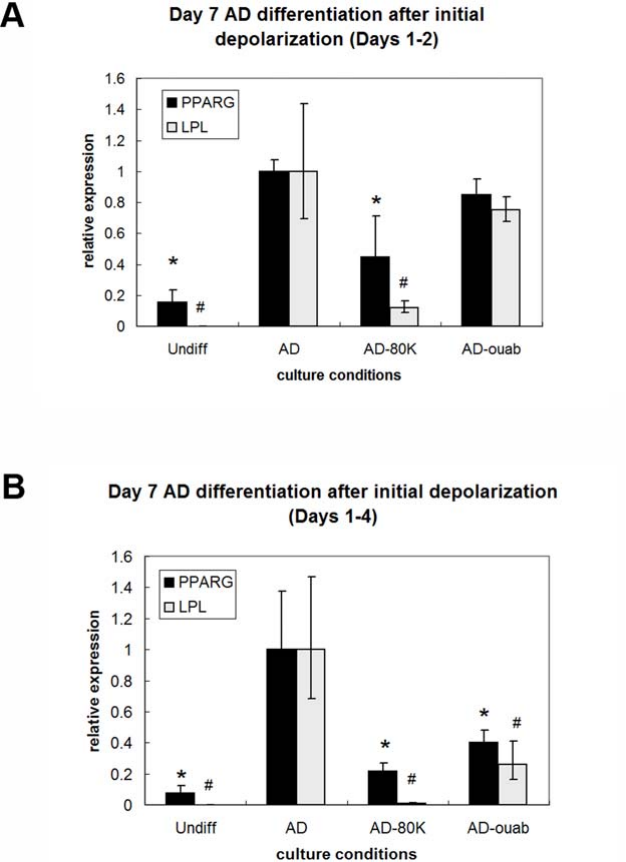
Shorter, earlier depolarization times are sufficient to suppress AD differentiation. Cells were exposed to 80 mM K^+^ (AD-K) or 10 nM ouabain (AD-ouab) during Days 1–2 (A) or Days 1–4 (B), then washed and continued in culture in AD medium. Gene expression was evaluated on Day 7. Two days of exposure to 80 mM K^+^ or four days of exposure to 10 nM ouabain was sufficient to effect a change in AD marker expression. Data points are mean relative expression±standard deviation (N = 6). Marked samples are statistically different, * relative to PPARG expression of untreated AD samples (p<0.002), # relative to LPL expression of untreated AD samples (p<0.002). Undiff, hMSCs cultured in control medium; AD, hMSCs cultured in AD medium; AD-80K, hMSCs cultured in AD medium supplemented with 80 mM K^+^; AD-ouab, hMSCs cultured in AD medium supplemented with 10 nM ouabain.

**Figure 6:**
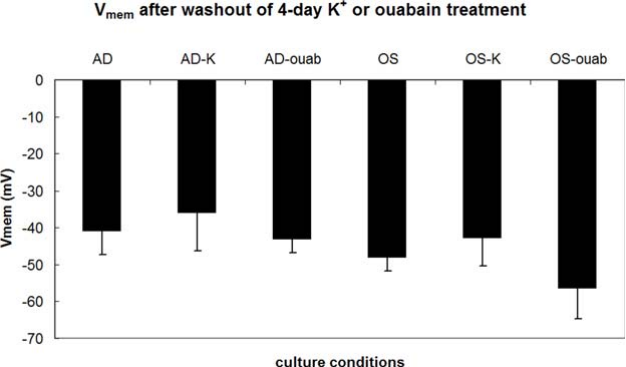
Membrane potential of AD and OS cells recovers after washout of early depolarization treatments. hMSCs in AD or OS differentiation media were depolarized with 80 mM K^+^ (AD-K, OS-K) or 10 nM ouabain (AD-ouab, OS-ouab) during Days 1–4. Control cells were cultured in normal AD or OS media (AD or OS). Depolarization treatment was washed out after Day 4 and replaced with normal AD or OS media. Intracellular recordings were performed after washout on Days 5 or 6. Data points are mean potentials±standard deviation (N = 7–10 cells). Neither treated AD cells nor treated OS cells were statistically different from their respective untreated controls (p<0.05).

To better understand the temporal effects of depolarization on the progression of AD differentiation, K^+^ and ouabain treatments were administered early during culture (Days 1–2 or Days 1–4), then washed out and replaced with AD medium. Cells were cultured until Day 7, and gene expression was evaluated to determine whether the shorter exposure time was sufficient to change expression patterns (Fig. 5). Exposure to 10 nM ouabain for two days did not significantly change PPARG or LPL expression (Fig. 5A), but a four-day exposure resulted in a 2.5-fold decrease in PPARG and 3.7-fold decrease in LPL expression (Fig. 5B, p<0.02) compared to untreated AD. In contrast, exposure to 80 mM K^+^ for two days decreased gene expression levels (2.1- and 8.5-fold for PPARG and LPL, respectively, Fig. 5A, p<0.02) compared to untreated AD cells. Four-day exposure to K^+^ further decreased LPL levels dramatically by 113.0-fold (Fig. 5B, p<0.009). When the K^+^ and ouabain treatments were removed after four days, electrophysiology measurements showed that the V_mem_ of the cells recovered to similar levels as untreated AD cells by Day 5 (Fig. 6). The ability of the depolarization treatments to suppress AD-related gene expression after washout despite recovery of V_mem_ suggests that the instructive V_mem_ signal acts early during the differentiation process and is thus unaffected by the subsequent recovery of normal V_mem_ levels. It is important to note that the V_mem_ recordings of untreated AD cells in Fig. 6 cannot be compared to the V_mem_ values extrapolated from the voltage dye studies in Table 1 because of differences in culture conditions. The intracellular recordings were acquired from hMSCs that had been cultured and differentiated for a total of five days, whereas the voltage dye studies were conducted after a total of four weeks of culture, with differentiation being initiated at various time points within those four weeks.

### Depolarization inhibits OS differentiation

After characterizing the AD response to depolarization, we sought to determine whether the observed suppression of differentiation is restricted to AD lineages only, or whether control of V_mem_ is an important parameter for other differentiation trajectories as well. To address this question, we induced depolarization during OS differentiation using the same approach as with AD differentiation. Since both OS and AD differentiation normally exhibit hyperpolarization, our hypothesis was that just as in AD differentiation, the normal progression of OS differentiation would be inhibited by externally induced depolarization.

Extent of OS differentiation was determined by quantifying the gene expression levels of alkaline phosphatase (ALP) and bone sialoprotein (BSP), the activity of ALP, and the calcium content of the depolarized cells. ALP is a membrane-bound glycoprotein that plays a role in early osteogenesis and also initiates matrix mineralization [38]. BSP is a secreted glycoprotein mainly localized to the extracellular matrix of bone tissue [39]. It is associated temporally and spatially with calcification events during later stages of osteogenesis and acts as a nucleator for hydroxyapatite crystal formation, thus facilitating bone mineralization [40], [41]. The resulting tissue has a high concentration of calcium ions, as the inorganic mineral phase of bone consists mainly of calcium hydroxyapatite [42].

### Depolarization by both high [K^+^]_out_ and ouabain suppresses OS differentiation

**Figure 7:**
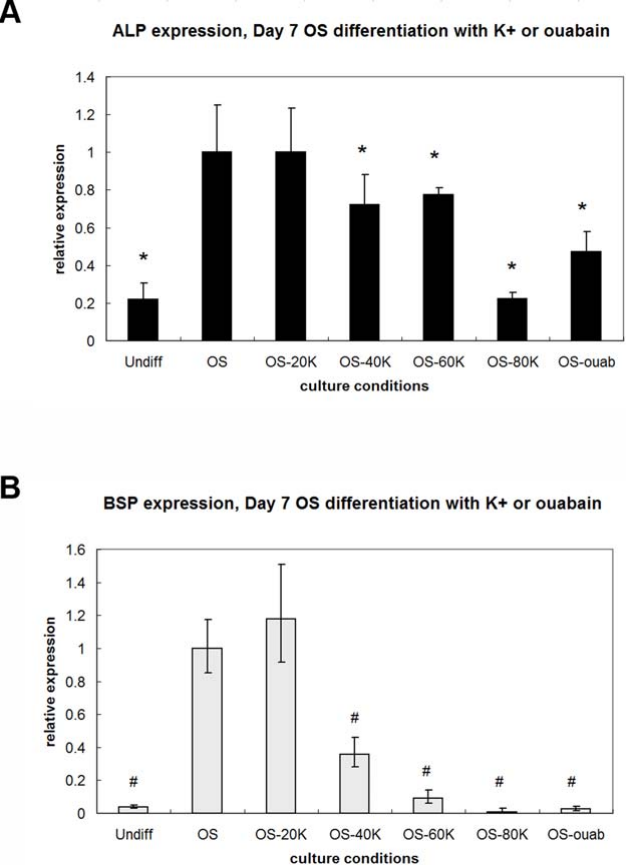
Depolarization suppresses OS gene expression. Expression of ALP (A) and BSP (B) decreased by Day 7 of OS differentiation in response to addition of 40–80 mM K^+^ (OS-40K, OS-60K, OS-80K) or 10 nM ouabain (OS-ouab). Data points are mean relative expression±standard deviation (N = 6). Marked samples are statistically different, * relative to ALP expression of untreated OS samples (p<0.04), # relative to BSP expression of untreated OS samples (p<0.002). Undiff, hMSCs cultured in control medium; OS, hMSCs cultured in OS medium; OS-20K, OS-40K, OS-60K, OS-80K, hMSCs cultured in OS medium supplemented with 20, 40, 60, 80 mM K^+^, respectively; OS-ouab, hMSCs cultured in OS medium supplemented with 10 nM ouabain.

**Figure 8:**
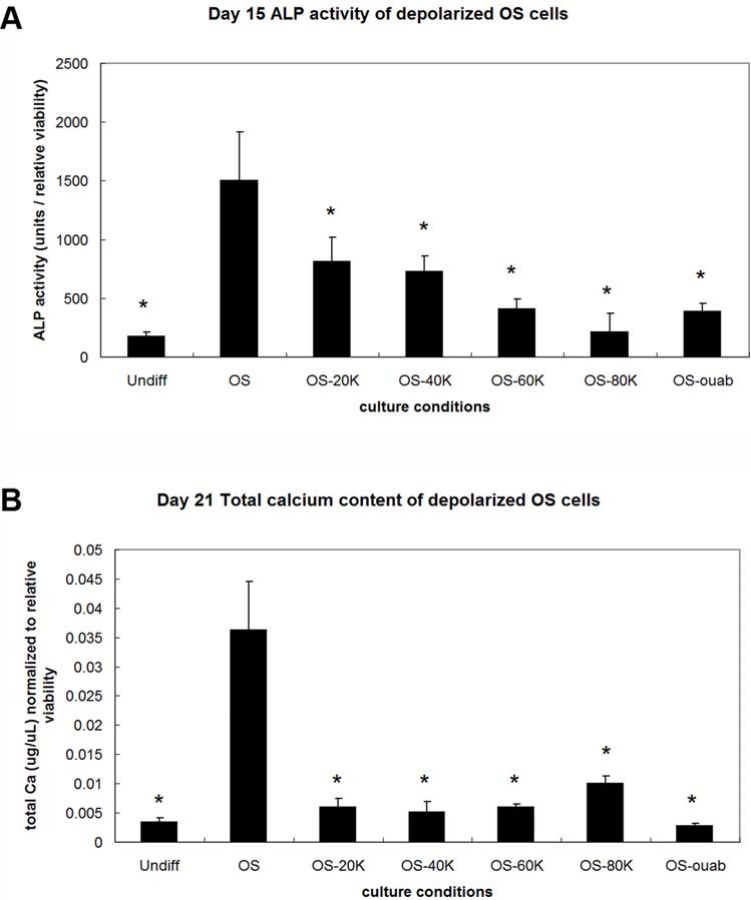
Depolarization suppresses ALP activity and reduces calcium content during OS differentiation. (A) ALP activity decreased during OS differentiation in cells treated with 20–80 mM K^+^ (OS-20K, OS-40K, OS-60K, OS-80K) or 10 nM ouabain (OS-ouab). Data points are mean ALP activity units normalized to relative cell viability±standard deviation (N = 6). Marked samples are statistically different * relative to untreated OS samples (p<0.008). Undiff, hMSCs cultured in control medium; OS, hMSCs cultured in OS medium; OS-20K, OS-40K, OS-60K, OS-80K, hMSCs cultured in OS medium supplemented with 20, 40, 60, 80 mM K^+^, respectively; OS-ouab, hMSCs cultured in OS medium supplemented with 10 nM ouabain. (B) Total calcium content of cells undergoing OS differentiation was lowered by addition of 20–80 mM K^+^ (OS-20K, OS-40K, OS-60K, OS-80K) or 10 nM ouabain (OS-ouab). Data points are mean calcium content normalized to relative cell viability±standard deviation (N = 6). Marked samples are statistically different * relative to untreated OS samples (p<0.002). Undiff, hMSCs cultured in control medium; OS, hMSCs cultured in OS medium; OS-20K, OS-40K, OS-60K, OS-80K, hMSCs cultured in OS medium supplemented with 20, 40, 60, 80 mM K^+^, respectively; OS-ouab, hMSCs cultured in OS medium supplemented with 10 nM ouabain.

Treatment with both 40–80 mM K^+^ and 10 nM ouabain suppressed OS differentiation by Day 7. Ouabain treatment decreased ALP by 2.1-fold and BSP by 34.9-fold (p<0.002, Fig. 7A,B). Treatment with 40, 60, and 80 mM K^+^ decreased ALP by 1.4-, 1.3-, and 4.6-fold, respectively, and BSP by 2.7-, 10.3-, and 69.8-fold, respectively (p<0.002), compared to untreated OS samples (Fig. 7A,B). Alkaline phosphatase (ALP) activity on Day 15 (Fig. 8A) and calcium content on Day 21 (Fig. 8B) were also measured as indicators of bone matrix formation and mineralization, respectively. Ouabain-treated cells had 3.9-fold decreased ALP activity compared to untreated OS cells (Fig. 8A, p<0.001). Similarly, K^+^-treated OS cells exhibited decreased ALP activity in a dose-dependent manner (1.8, 2.1, 3.7, 7.1-fold decreased activity for increasing K^+^ concentrations, Fig. 8A, p<0.008). Calcium content on Day 21 was lower for all depolarized samples compared to untreated OS cells, with ouabain treatment yielding the least calcium content (12.8-fold decrease, Fig. 8B, p<0.0009) and K^+^ treatment yielding non-dose-dependent decreases in calcium content (6.1-, 7.1-, 6.1-, and 3.6-fold decreases with increasing K^+^ concentrations, Fig. 8B, p<0.002).

### Shorter durations of depolarization are sufficient to suppress OS differentiation

**Figure 9:**
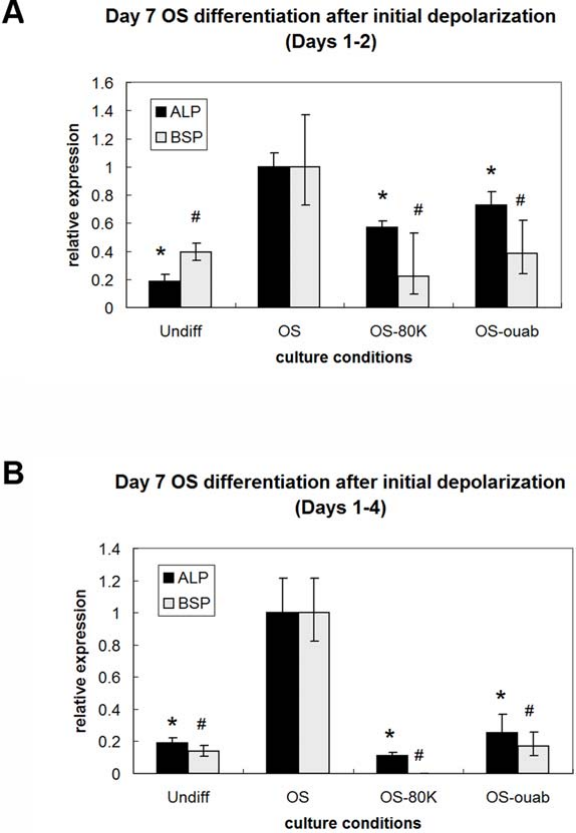
Shorter, earlier depolarization times are sufficient to suppress OS differentiation. Cells were exposed to 80 mM K^+^ or 10 nM ouabain during Days 1–2 (A) or Days 1–4 (B), then washed and continued in culture in OS medium. Gene expression was evaluated on Day 7. Two or four days of exposure to depolarization treatment was sufficient to effect a change in OS marker expression. Data points are mean relative expression±standard deviation (N = 6). Marked samples are statistically different * relative to ALP expression of untreated OS samples (p<0.009), # relative to BSP expression of untreated OS samples (p<0.04). Undiff, hMSCs cultured in control medium; OS, hMSCs cultured in OS medium; OS-80K, hMSCs cultured in OS medium supplemented with 80 mM K^+^; OS-ouab, hMSCs cultured in OS medium supplemented with 10 nM ouabain.

The duration of K^+^ exposure or ouabain exposure was shortened to determine whether depolarization-induced changes differed with treatment duration. Both treatments (80 mM K^+^ and 10 nM ouabain) decreased OS-related gene expression after an initial two-day exposure (p<0.04, Fig. 9A, left). In addition, as with AD differentiation, a four-day exposure to the treatments resulted in larger decreases in both ALP expression (3.8- and 9.2-fold for ouabain and K^+^, respectively, p<0.0001) and BSP expression by Day 7 (5.7-fold decrease for ouabain, p<0.0004, and undetectable levels for K^+^) (Fig. 9B, right). Although the suppression of OS-related gene expression persisted after washout of K^+^ or ouabain treatments, V_mem_ recovered to similar values as that of untreated OS cells (Fig. 6). Altogether, these data show that two days of early depolarization can inhibit differentiation as revealed by reduced expression of OS markers, and four days of early depolarization makes these changes even more apparent. These changes in phenotype persist despite recovery of normal V_mem_ after washout of early depolarization treatments.

### Hyperpolarization during OS differentiation causes upregulation of osteogenic markers

**Figure 10:**
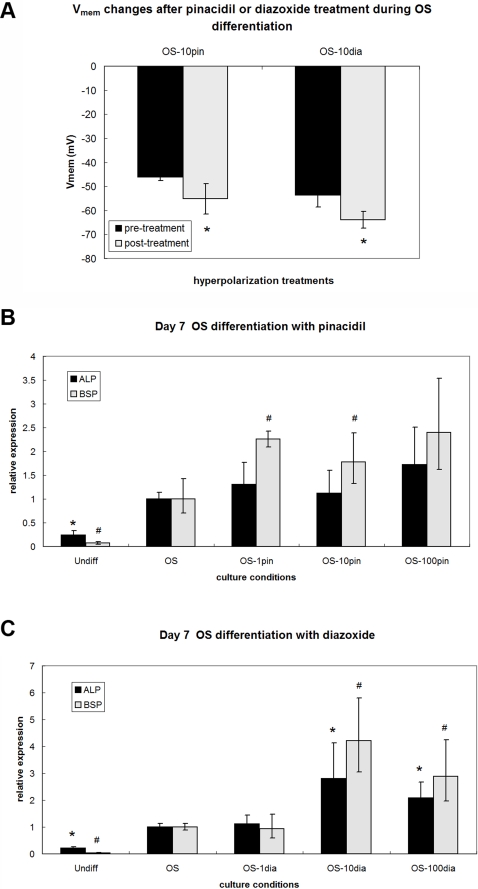
Hyperpolarization upregulates OS gene expression. (A) K-_ATP_-channel openers pinacidil and diazoxide hyperpolarized hMSCs undergoing OS differentiation. Cells were impaled individually and the V_mem_ recorded until a stable baseline was reached (pre-treatment), then 10 µM pinacidil or diazoxide was added and the V_mem_ recorded until a new equilibrium was reached (post-treatment). Data points are mean potentials±standard deviation (N = 5 cells). Marked samples are statistically different * relative to respective pre-treatment samples (p<0.04). (B, C) Exposure to K-_ATP_-channel openers pinacidil (B) and diazoxide (C) resulted in slight upregulation of OS markers compared to untreated cells. When treated with 1 and 10 µM pinacidil (OS-1pin and OS-10pin, respectively), cells showed upregulated BSP expression compared to untreated OS cells (p<0.04). When treated with 10 and 100 µM diazoxide, cells upregulated ALP and BSP expression compared to untreated OS cells. Data points are mean relative expression±standard deviation (N = 6). Marked samples are statistically different * relative to ALP expression of untreated OS samples (p<0.05), # relative to BSP expression of untreated OS samples (p<0.05). Undiff, hMSCs cultured in control medium; OS, hMSCs cultured in OS medium; OS-80K, hMSCs cultured in OS medium supplemented with 80 mM K^+^; OS-ouab, hMSCs cultured in OS medium supplemented with 10 nM ouabain.

The above depolarization studies show that depolarization prevents the normal differentiation of hMSC cells in two lineages. To rule out non-specific effects of depolarization and determine whether control of transmembrane potential is an instructive, bi-directional determinant of differentiation trajectory, we next asked whether hyperpolarization affected differentiation. If V_mem_ is a true determinant of differentiation potential, as has been suggested [43], hyperpolarization ought to have the reverse effect of depolarization and thus induce differentiation. OS-differentiating cells were exposed to K_ATP_ channel openers pinacidil and diazoxide (1, 10, 100 µM), reagents known to hyperpolarize various cell types [44]–[47]. Intracellular recordings confirmed that hMSCs exposed to 10 µM pinacidil or diazoxide within the first four days of OS differentiation indeed hyperpolarized by 9 mV with pinacidil treatment (p<0.04) and by 10 mV with diazoxide treatment (p<0.03, Fig. 10A). Gene expression of hyperpolarized OS cells was evaluated relative to untreated OS cells after 7 days. Exposure to 1 µM and 10 µM pinacidil resulted in a 1.8-fold and 2.2-fold increase in BSP expression, respectively (Fig. 10B, p<0.04), but no significant change in ALP expression. Exposure to 10 µM and 100 µM diazoxide caused upregulation of ALP expression by 2.9-fold and 2.1-fold, respectively, and upregulation of BSP expression by 4.3-fold and 3.1-fold, respectively (Fig. 10C, p<0.05). Thus, hyperpolarization of differentiating OS cells by pinacidil and diazoxide exposure elevates bone marker expression. These results suggest that depolarization is not merely permissive for differentiation, but rather that V_mem_ is a signal whose value functionally determines the differentiation propensity of hMSCs.

## Discussion

In this study, the characteristic membrane potential profiles for hMSCs during OS and AD differentiation were obtained using the voltage-sensitive fluorescent dye DiSBAC_2_(3). Fluorescence readings of differentiating cells decreased in intensity as a function of differentiation time, which corresponds to more negative membrane potential, or hyperpolarization. Hyperpolarization occurred during both OS and AD differentiation: based on the measured voltage-dependent fluorescence changes, we estimated that V_mem_ hyperpolarized by a net change of 56 and 74 mV after 4 weeks of OS and AD differentiation, respectively. These trends agree with several comparative analyses in the literature that examine V_mem_ and ion flux in various cell types and suggest a relationship between depolarization and control of differentiation and proliferation [1], [43], [48]–[50]. According to these reports, proliferative and relatively immature cells (such as those of the developing embryo) exhibit strongly depolarized membrane potentials, while terminally differentiated quiescent cells exhibit strongly hyperpolarized membrane potentials. Our observation that the process of differentiation was accompanied by a corresponding hyperpolarization in membrane potential is consistent with these data; as the stem cells are moving from their undifferentiated state to a committed phenotype, their membrane potentials shift from values that are characteristic of developing cells towards levels observed in terminally-differentiated, committed, somatic cells.

V_mem_ changes and other electrophysiological properties are traditionally studied in excitable cells such as neurons and myoblasts, rather than the non-excitable cells studied here. Interestingly, precursors of excitable cell types exhibit similar V_mem_ hyperpolarization during development and commitment compared to what we have seen in OS- and AD-differentiated hMSCs. For example, maturation of neuroblastoma cells derived from neural crest cells can be characterized by an ordered expression of currents: a human *eag*-related K^+^ channel current and a delayed rectifier K^+^ channel current are detected at early stages and are and replaced at later stages by a tetrodotoxin-sensitive Na^+^ channel current and an inward rectifier K^+^ channel current [21], [51], [52]. The changes in current cause the cells to undergo a net V_mem_ hyperpolarization during maturation. Similarly, the differentiation and fusion of myoblasts to form myotubes is accompanied by V_mem_ hyperpolarization from −10 to −70 mV [53], [54]. Kir2.1 channel activity, which is responsible for this hyperpolarization, initiates myogenesis by triggering the expression of early transcription factors such as myogenin and myocyte enhancer factor (MEF2) [27]. Therefore, hyperpolarization plays an important role in differentiation and maturation of both excitable and non-excitable cell types. In light of recent work exploring the differentiation potential of hMSCs beyond the OS, AD, and chondrogenic lineages, it would be interesting to compare V_mem_ changes in neural or myogenic hMSC differentiation to the hyperpolarization reported in neural precursors, myoblasts, and OS- and AD-differentiated hMSCs. Furthermore, if hyperpolarization does indeed occur during stem cell differentiation into neural precursors and myoblasts, V_mem_ modulation could be an attractive way to improve differentiation into these excitable cell types.

To determine whether hyperpolarization is functionally necessary for OS and AD differentiation, we disrupted the membrane potential of differentiating hMSCs and studied the subsequent changes in tissue-specific phenotypes. Membrane potential disruption (depolarization) was achieved by two classic methods: addition of high concentrations of extracellular K^+^ (high [K^+^]_out_), or addition of the Na^+^/K^+^ ATPase blocker ouabain. We used two independent methods to ensure that the effect was due specifically to reduction of transmembrane potential, and not side-effects of the individual treatments.

Addition of 80 mM [K^+^] to the differentiation medium resulted in marked suppression of the AD markers PPARG and LPL on Day 7 compared to untreated cells. This suppression of AD gene expression continued through Day 22. Lower concentrations of extracellular K^+^ did not induce the same changes in AD gene expression (data not shown). The different behavior of cells in low vs. high [K^+^]_out_ in an AD-inducing environment suggests that there is an optimal [K^+^]_out_ at which the resulting depolarization effects a change in AD phenotype, rather than a range of [K^+^]_out_ over which a graded response can be observed. During OS differentiation however, Day 7 expression of the mature bone marker BSP varied in a graded, does-dependent manner, with 20 mM [K^+^]_out_ having no significant effect and 80 mM [K^+^]_out_ having the greatest effect. The differences in AD and OS cell response to different high [K^+^]_out_ concentrations indicate that progression toward these two lineages changes the cells' sensitivities to different V_mem_ levels and their tolerance levels with respect to depolarization. Whereas AD cells have a higher tolerance for V_mem_ changes and show no response below 80 mM [K^+^]_out_, OS cells have a lower tolerance and respond proportionately to changes of 40 mM or more. Thus, not only is hMSC differentiation disrupted by depolarization, but more specifically, commitment to specific lineages (AD or OS) can be characterized by unique sensitivities to V_mem_ changes.

Addition of the Na^+^/K^+^ ATPase blocker ouabain (10 nM) also caused suppression of differentiation markers. Ouabain treatment significantly suppressed both AD and OS gene expression, although the fold-changes are smaller than to those seen with high [K^+^]_out_ treatment. During OS differentiation, ALP activity and calcium content were also significantly reduced. In contrast to the effects of two treatments that depolarize cells by different mechanisms, bone-related gene expression was upregulated in response to hyperpolarizing K^+^ channel openers. Because hyperpolarization treatments caused an opposite effect to the depolarization treatments (depolarization suppresses differentiation, while hyperpolarization increases differentiation), our data support a view of membrane potential as an instructive determinant of differentiation state.

Washout experiments showed that early but short exposure to high [K^+^]_out_ was sufficient to cause similar decreases in tissue-related gene expression as constant exposure. Exposure for the first two days or the first four days of culture (29% and 57% of culture time, respectively) resulted in decreased fat- and bone-related gene expression by Day 7. As with high [K^+^]_out_ treatment, the effects of ouabain on gene expression can be seen with early but short exposure. An initial exposure of 2 days was sufficient to reduce OS gene expression, and an initial exposure of 4 days was sufficient to reduce AD gene expression. Interestingly, suppression of the differentiated phenotype was observed despite the recovery of V_mem_ to normal levels after washout of depolarization treatment. This suggests that the signal mediated by V_mem_ hyperpolarization is transduced to downstream controls of cell behavior during early stages of differentiation.

It is important to note that the differentiation markers assessed in the above studies are bone- and fat-specific, and do not simply reflect general cell viability and health. In response to V_mem_ depolarization, cells exhibited mostly decreased OS and AD marker expression compared to the normal trajectory of OS and AD differentiation. However, when compared to undifferentiated hMSCs, the depolarized cells consistently showed elevated OS or AD marker expression. The fact that the cells did initiate lineage-specific gene and metabolite production indicates that this cell response cannot be simply due to stress. Rather, the depolarized cells started the differentiation process but were restricted from reaching a fully differentiated phenotype, due to instructive signals provided by the changes in V_mem_. Furthermore, gene expression of some markers was increased in depolarized cells even compared to untreated differentiated cells. For example, PPARG expression of ouabain-treated AD cells was almost 2-fold greater than that of untreated AD cells on Day 2 (Fig. 4), implying an increase in transcriptional activity not characteristic of stressed cells. Finally, the discovery that OS differentiation can not only be suppressed by depolarization, but can also be augmented with hyperpolarization, strongly suggests that the V_mem_ exerts specific control over differentiation events.

Taken together, these depolarization studies suggest that control of the endogenous V_mem_ is necessary for normal differentiation of hMSCs into bone and fat. Depolarization by addition of high [K^+^]_out_ or ouabain results in suppression of the differentiated phenotype. Furthermore, depolarization appears to exert its effect early in the differentiation process. Importantly, this effect is not due to toxicity or non-specific stress response because specific marker gene expression was induced by depolarization and hyperpolarization. The ability to either increase or decrease the differentiation propensity by controlled modulation of V_mem_ suggests strongly that, as proposed previously [43], [48], [55], [56], transmembrane potential level is an instructive signal controlling important aspects of cellular plasticity.

Our data extend the previous analyses of somatic vs. embryonic/cancer cell membrane voltage [1], [43], [49] to stem cells. Crucially, our functional results show that the relationship between depolarization and an undifferentiated state is not merely a correlation but rather that transmembrane potential is a functional determinant of differentiation state in hMSCs. Moreover, in complement to recent studies implicating specific ion channels in cell behavior, our data implicate V_mem_ as the salient parameter, not specific ion gradients or ion-independent functions of specific proteins. This is important for biomedical applications because it suggests that rational changes in cell behavior can be induced by techniques that do not depend on specific channel or pump genes being expressed natively in a cell population. A number of mechanisms have been proposed to explain how V_mem_ levels are transduced into transcriptional cascades by second messenger systems (reviewed in [1]), and we are currently focused on testing Ca^2+^ influx, voltage sensor-containing phosphatase (VSP)-PTEN transduction, and other mechanisms in the voltage control of hMSC differentiation. It clear, however, that control of bioelectrical properties of stem cells is not only a fascinating fundamental aspect of cell regulation, but also potentially a useful tool in tissue engineering efforts. Future work in regenerative medicine may be able to capitalize on V_mem_ as a powerful and tractable control point for rational modulation of adult stem cell function.

## Materials and Methods

### hMSC cultivation

hMSCs were obtained from bone marrow aspirates from Cambrex Bio Science Walkersville, Inc., from a 25 yr old healthy male and prepared as we have previously reported [57]. Whole bone marrow aspirates were plated at a density of 10 µL of aspirate per centimeter squared in 185 cm^2^ tissue culture flasks in Dulbecco's Modified Eagle Medium (DMEM) supplemented with 10% fetal bovine serum (FBS), penicillin (100 U/mL), streptomycin (100 µg/mL), 0.1 mM non-essential amino acids, and basic fibroblast growth factor (bFGF, 1 ng/mL) (Invitrogen, Carlsbad, CA). Cells were maintained in a humidified incubator at 37°C with 5% CO_2_. hMSCs were separated from hematopoietic stem cells on the basis of their adherence to tissue culture plastic; hematopoietic stem cells in suspension were removed after approximately 5 days of culture. hMSCs were expanded until confluent, with medium changes every 3–4 days. Cells were trypsinized with 0.25% trypsin-1mM EDTA (Invitrogen), and frozen in liquid nitrogen in FBS with 10% DMSO as first passage cells (P1). P1-P3 cells were used for all experiments.

### Differentiation

hMSCs were thawed and plated on glass bottom dishes or on tissue culture polystyrene at a density of 5000 cells/cm^2^ for osteogenic (OS) differentiation or 10000 cells/cm^2^ for adipogenic (AD) differentiation. For the first 24 hr post-thaw, cells were cultured in control medium, consisting of DMEM supplemented with 10% FBS, penicillin (100 U/mL), streptomycin (100 µg/mL), and 0.1 mM non-essential amino acids. Differentiation inducers were then added to the medium in order to study temporal-dependent changes in membrane potential during OS and AD differentiation. Control medium was supplemented with 10 mM β-glycerophosphate, 0.05 mM L-ascorbic acid-2-phosphate, and 100 nM dexamethasone for OS differentiation; or 0.5 mM 3-isobutyl-1-methyl-xanthine, 1 µM dexamethasone, 5 µg/mL insulin, and 50 µM indomethacin for AD differentiation (Sigma-Aldrich, St. Louis, MO). Undifferentiated hMSCs were maintained in control medium.

### Disruption of membrane potential

To assess the effects of disruption of membrane potential (V_mem_), several methods were employed to change V_mem_. (1) Na^+^/K^+^-ATPase-inhibitor ouabain (10 nM, Sigma-Aldrich) was added to the medium from a fresh 10 mM stock solution in distilled water. (2) The concentration of extracellular K^+^ was increased by adding potassium gluconate (Sigma-Aldrich) to the medium to final concentrations of 10–80 mM. (3) The ATP-sensitive potassium channel (K_ATP_ ) openers pinacidil and diazoxide (Sigma-Aldrich) were added to the medium to final concentrations of 1, 10, or 100 µM from 10 mM stock solutions in ethanol.

### Confocal imaging using voltage-sensitive fluorescent dyes

After 0, 1, 2, 3, or 4 weeks of AD or OS differentiation (culture schedule presented in Fig. 1A), cells were dyed with a fluorescent dye that is sensitive to membrane potential. Bis-(1,3-diethylthiobarbituric acid)trimethine oxonol (DiSBAC_2_(3) or DiSBAC, Invitrogen) is an anionic voltage-sensitive dye whose uptake into cells is voltage-dependent: higher uptake is seen in more depolarized cells. A fresh solution of 10 mM DiSBAC in DMSO was prepared and diluted to 0.5 µM in Hank's Buffered Salt Solution (HBSS, Invitrogen). Cells grown in glass-bottom dishes (poly-d-lysine coated, No. 1.5, MatTek Corp., Ashland, MA) were incubated in DiSBAC for 30 minutes at 37°C, then imaged while submerged in dye at room temperature. Images were acquired on a Leica TCS SP2 laser scanning confocal microscope with an inverted DM IRE2 stand (Wetzlar, Germany) and a Leica PL APO 63× (NA 1.2) water-immersion objective. DiSBAC was excited with a 543 nm HeNe laser; images were collected at 570±5 nm by a non-descanned PMT controlled by Leica Confocal Software. A double dichroic filter was used to eliminate 543 nm excitation light. Confocal images for all samples in an experimental set were taken on the same day to minimize instrumental and other variations. Since fluorescence intensity was quantified for each image, the gain and offset settings of the microscope were kept constant over the duration of each experiment. To visualize membrane potential depolarization, cells at resting potential were imaged as above, then exposed to depolarization agents and allowed to equilibrate for 5 min, then imaged once more. MATLAB software (The MathWorks, Inc.) was used to assist in the drawing of regions of interest (ROI) around cells and in calculating pixel intensities within the ROIs. ROIs were drawn on thresholded images by using the function bwboundaries to trace cells and their nuclei. Fluorescence intensities of cells encircled by ROIs were calculated by averaging corresponding pixel intensities in the original image, excluding pixels within encircled nuclei, after background correction using a blank (no cell) region of the image.

### Intracellular recordings

Membrane potentials of hMSCs were recorded during OS and AD differentiation before and after depolarization treatments. Electrodes were pulled from borosilicate glass capillary tubing (1.0 mm OD, Warner Instrument Corp., Hamden, CT) using a Flaming/Brown Micropipette Puller (Model P-87, Sutter Instrument Co., Novato, CA), filled with a filtered 2 M KCl electrode solution, and loaded onto a Burleigh PCS PS60 micromanipulator (EXFO Life Sciences, Mississauga, Ontario). Electrode resistances were in the range of 40–60 MΩ. Signals were passed through a preamplifier (8100-1 Single Electrode System, Dagan Corp., Minneapolis, MN) and an amplifier (Instrumentation Amplifier Model 210, Brownlee Precision Co., San Jose, CA). Data was collected using a DI-720 Series #3 Data Acquisition System (Dataq Instruments, Inc., Akron, OH) controlled by WinDaq Waveform Recording Software, and was analyzed using WinDaq Waveform Browser (Dataq Instruments, Inc.). Individual cells were impaled, and membrane potential was recorded relative to the bath solution (HBSS, Invitrogen). After obtaining a stable signal, depolarizing or hyperpolarizing agents were added to the bath solution, and the membrane potential was recorded for an additional 5 min, or until the signal stabilized.

### RNA isolation, purification, and real time RT-PCR

RNA was isolated from hMSCs using Trizol reagent (Invitrogen) following the single step acid-phenol guanidinium method, and purified using the Qiagen RNEasy kit (Qiagen, Valencia, CA). To synthesize cDNA, reverse transcription was performed on the purified RNA using the High Capacity cDNA Archive kit (Applied Biosystems, Foster City, CA). Real time RT-PCR was performed on differentiated cells with and without depolarization treatment to track the expression of markers characteristic of the differentiated cell types. Osteoblast differentiation markers include alkaline phosphatase (ALP) and bone sialoprotein (BSP). Adipogenic differentiation markers include perixosome proliferator-activated receptor γ (PPARG) and lipoprotein lipase (LPL). Primers and probes for the bone-related and adipose-related genes above were obtained from TaqMan® Gene Expression Assay kits (Applied Biosystems). Transcript expression levels were quantified with an ABI Prism 7000 Real Time PCR System (Applied Biosystems) or a Stratagene Mx3000P QPCR System (Stratagene, La Jolla, CA). Expression levels were normalized to the housekeeping gene glyceraldehyde 3-phosphate dehydrogenase (GAPDH) and reported relative to positive control reactions (untreated OS or AD) [58]–[60]. We have previously reported PCR reaction conditions and primers [61]–[63].

### Calcium assay

Total calcium content was determined by a colorimetric assay using the Calcium (CPC) Liquicolor Test (Stanbio Laboratory, Boerne, TX). Calcium was dissociated with trichloroacetic acid and reacted with o-cresolphthalein complexone, forming a purple color which was measured spectrophotometrically at 575 nm using a microplate reader (VersaMax, Molecular Devices, Sunnyvale, CA).

### Alkaline phosphatase (ALP) assay

Enzyme activity was assessed using a biochemical assay from Sigma-Aldrich that detects the hydrolysis of p-nitrophenyl phosphate (pNPP) to p-nitrophenol by ALP. Cultured cells were lysed with 0.2% v/v Triton X-100 in 5 mM MgCl_2_, and incubated with pNPP substrate in 2-amino-2-methyl-1-propanol buffer. The hydrolysis reaction was stopped by 0.2 M NaOH, and the colored end product was detected spectrophotometrically at 405 nm.

### Oil Red O Staining

Cells undergoing AD differentiation were stained with Oil Red O to visualize lipid droplet accumulation. Cells were fixed overnight in 4% neutral buffered formalin, then washed with 60% isopropanol and air-dried. A fresh 60% Oil Red O working solution was prepared from a stock solution (0.7 g Oil Red O in 200 mL isopropanol) and filtered through a 45 µM syringe filter. Cells were stained with the working solution for 45 min, washed five times with distilled water, and imaged at room temperature with an inverted microscope (Axiovert S100, Carl Zeiss, Inc.) equipped with Zeiss A-Plan 10× (NA 0.25) and LD A-Plan 32× (0.40) objectives. Images captured by a Sony Exwave HAD CCD camera were acquired using ImageJ software (NIH). Photoshop software (Adobe Systems Inc.) was used to adjust levels and color balance. To measure the amount of Oil Red O in the stained samples, Oil Red O was eluted from the cells by adding 100% isopropanol and incubating for 1 hr, and then transferred to a 96-well plate and measured spectrophotometrically at 500 nm.

### Cell viability

Cell viability was assessed using the alamarBlue assay (Invitrogen). Cells were incubated at 37°C in a solution of 3% alamarBlue in control medium, and the resulting fluorometric change was detected with an excitation wavelength of 560 nm and emission wavelength of 590 nm using a microplate spectrofluorometer (SpectraMax Gemini EM, Molecular Devices, Sunnyvale, CA).

### Statistics

Statistically significant differences between groups were determined by performing a two-tailed Student's t-test. Differences were considered significant if p<0.05, unless otherwise noted.

## References

[1] Levin M (2007). Large-scale biophysics: ion flows and regeneration.. Trends in Cell Biology.

[2] McCaig CD, Rajnicek AM, Song B, Zhao M (2005). Controlling cell behavior electrically: Current views and future potential.. Physiological Reviews.

[3] Altizer AM, Moriarty LJ, Bell SM, Schreiner CM, Scott WJ (2001). Endogenous electric current is associated with normal development of the vertebrate limb.. Developmental Dynamics.

[4] Altizer AM, Stewart SG, Albertson BK, Borgens RB (2002). Skin flaps inhibit both the current of injury at the amputation surface and regeneration of that limb in newts.. Journal of Experimental Zoology.

[5] Borgens RB, Callahan L, Rouleau MF (1987). Anatomy of axolotl flank integument during limb bud development with special reference to a transcutaneous current predicting limb formation.. Journal of Experimental Zoology.

[6] Borgens RB, Vanable JW, Jaffe LF (1977). Bioelectricity and regeneration: Large currents leave the stumps of regenerating newt limbs.. Proceedings of the National Academy of Sciences of the United States of America.

[7] Borgens RB, Vanable JW, Jaffe LF (1979). Reduction of sodium dependent stump currents disturbs urodele limb regeneration.. Journal of Experimental Zoology.

[8] Jenkins LS, Duerstock BS, Borgens RB (1996). Reduction of the current of injury leaving the amputation inhibits limb regeneration in the red spotted newt.. Developmental Biology.

[9] Nuccitelli R (2003). Endogenous electric fields in embryos during development, regeneration and wound healing.. Radiation Protection Dosimetry.

[10] Becker RO, Spadaro JA (1972). Electrical stimulation of partial limb regeneration in mammals.. Bulletin of the New York Academy of Medicine: Journal of Urban Health.

[11] Borgens RB, Vanable JW, Jaffe LF (1977). Bioelectricity and regeneration. I. Initiation of frog limb regeneration by minute currents.. Journal of Experimental Zoology.

[12] Smith SD (1974). Effects of electrode placement on stimulation of adult frog limb regeneration.. Annals of the New York Academy of Sciences.

[13] Adams DS, Masi A, Levin M (2007). H+ pump-dependent changes in membrane voltage are an early mechanism necessary and sufficient to induce Xenopus tail regeneration.. Development.

[14] Putney LK, Barber DL (2003). Na-H Exchange-dependent Increase in Intracellular pH Times G2/M Entry and Transition.. Journal of Biological Chemistry.

[15] Erskine L, McCaig CD (1995). Growth cone neurotransmitter receptor activation modulates electric field-guided nerve growth.. Developmental Biology.

[16] McCaig CD, Erskine L, Anonymous (1996). Nerve growth and nerve guidance in a physiological electric field.. Nerve Growth and Nerve Guidance.

[17] McCaig CD, Sangster L, Stewart R (2000). Neurotrophins enhance electric field-directed growth cone guidance and directed nerve branching.. Developmental Dynamics.

[18] Stewart R, Erskine L, McCaig CD (1995). Calcium channel subtypes and intracellular calcium stores modulate electric field-stimulated and -oriented nerve growth.. Developmental Biology.

[19] Zhao M, Song B, Pu J, Wada T, Reid B (2006). Electrical signals control wound healing through phosphatidylinositol-3-OH kinase-γ and PTEN.. Nature.

[20] Song B, Zhao M, Forrester JV, McCaig CD (2002). Electrical cues regulate the orientation and frequency of cell division and the rate of wound healing in vivo.. Proceedings of the National Academy of Sciences of the United States of America.

[21] Biagiotti T, D'Amico M, Marzi I, Di Gennaro P, Arcangeli A (2006). Cell renewing in neuroblastoma: Electrophysiological and immunocytochemical characterization of stem cells and derivatives.. Stem Cells.

[22] Cai J, Cheng A, Luo Y, Lu C, Mattson MP (2004). Membrane properties of rat embryonic multipotent neural stem cells.. Journal of Neurochemistry.

[23] Gersdorff Korsgaard MP, Christophersen P, Ahring PK, Olesen SP (2001). Identification of a novel voltage-gated Na+ channel rNav1.5a in the rat hippocampal progenitor stem cell line HiB5.. Pflugers Archiv European Journal of Physiology.

[24] Heubach JF, Graf EM, Leutheuser J, Bock M, Balana B (2004). Electrophysiological properties of human mesenchymal stem cells.. Journal of Physiology.

[25] Van Kempen MJA, Van Ginneken A, De Grijs I, Mutsaers NAM, Opthof T (2003). Expression of the electrophysiological system during murine embryonic stem cell cardiac differentiation.. Cellular Physiology and Biochemistry.

[26] Cho T, Bae JH, Choi HB, Kim SS, McLarnon JG (2002). Human neural stem cells: Electrophysiological properties of voltage-gated ion channels.. NeuroReport.

[27] Konig S, Hinard V, Arnaudeau S, Holzer N, Potter G (2004). Membrane hyperpolarization triggers myogenin and myocyte enhancer factor-2 expression during human myoblast differentiation.. Journal of Biological Chemistry.

[28] Sun W, Buzanska L, Domanska-Janik K, Salvi RJ, Stachowiak MK (2005). Voltage-sensitive and ligand-gated channels in differentiating neural stem-like cells derived from the nonhematopoietic fraction of human umbilical cord blood.. Stem Cells.

[29] Erdogan A, Schaefer CA, Schaefer M, Luedders DW, Stockhausen F (2005). Margatoxin inhibits VEGF-induced hyperpolarization, proliferation and nitric oxide production of human endothelial cells.. Journal of Vascular Research.

[30] Avram MM, Avram AS, James WD (2007). Subcutaneous fat in normal and diseased states. 3. Adipogenesis: From stem cell to fat cell.. Journal of the American Academy of Dermatology.

[31] Spiegelman BM (1998). PPAR-γ: Adipogenic regulator and thiazolidinedione receptor.. Diabetes.

[32] Chawla A, Schwarz EJ, Dimaculangan DD, Lazar MA (1994). Peroxisome proliferator-activated receptor (PPAR) γ: Adipose-predominant expression and induction early in adipocyte differentiation.. Endocrinology.

[33] Tontonoz P, Hu E, Graves RA, Budavari AI, Spiegelman BM (1994). mPPARγ2: Tissue-specific regulator of an adipocyte enhancer.. Genes and Development.

[34] Tontonoz P, Hu E, Spiegelman BM (1995). Regulation of adipocyte gene expression and differentiation by peroxisome proliferator activated receptor γ.. Current Opinion in Genetics and Development.

[35] Schoonjans K, Peinado-Onsurbe J, Lefebvre AM, Heyman RA, Briggs M (1996). PPARα and PPARγ activators direct a distinct tissue-specific transcriptional response via a PPRE in the lipoprotein lipase gene.. EMBO Journal.

[36] Schoonjans K, Staels B, Auwerx J (1996). The peroxisome proliferator activated receptors (PPARs) and their effects on lipid metabolism and adipocyte differentiation.. Biochimica et Biophysica Acta-Lipids and Lipid Metabolism.

[37] Auwerx J, Leroy P, Schoonjans K (1992). Lipoprotein lipase: Recent contributions from molecular biology.. Critical Reviews in Clinical Laboratory Sciences.

[38] Zur Nieden NI, Kempka G, Ahr HJ (2003). In vitro differentiation of embryonic stem cells into mineralized osteoblasts.. Differentiation.

[39] Fisher LW, McBride OW, Termine JD, Young MF (1990). Human bone sialoprotein. Deduced protein sequence and chromosomal localization.. Journal of Biological Chemistry.

[40] Hunter GK, Goldberg HA (1993). Nucleation of hydroxyapatite by bone sialoprotein.. Proceedings of the National Academy of Sciences of the United States of America.

[41] Hunter GK, Goldberg HA (1994). Modulation of crystal formation by bone phosphoproteins: Role of glutamic acid-rich sequences in the nucleation of hydroxyapatite by bone sialoprotein.. Biochemical Journal.

[42] Olszta MJ, Cheng X, Jee SS, Kumar R, Kim YY (2007). Bone structure and formation: A new perspective.. Materials Science and Engineering R: Reports.

[43] Binggeli R, Weinstein RC (1986). Membrane potentials and sodium channels: Hypotheses for growth regulation and cancer formation based on changes in sodium channels and gap junctions.. Journal of Theoretical Biology.

[44] Hoenicke EM, Damiano RJ (2001). Superior 12-hour heart preservation with pinacidil hyperpolarizing solution compared to University of Wisconsin solution.. Journal of Heart and Lung Transplantation.

[45] Lawson K (2000). Potassium channel openers as potential therapeutic weapons in ion channel disease.. Kidney International.

[46] Nielsen-Kudsk JE (1996). Potassium channel modulation: A new drug principle for regulation of smooth muscle contractility. Studies on isolated airways and arteries.. Danish Medical Bulletin.

[47] Xiong Y, Harmon CS (1995). Evidence that diazoxide promotes calcium influx in mouse keratinocyte cultures by membrane hyperpolarization.. Skin Pharmacology.

[48] Cone CD (1971). Unified theory on the basic mechanism of normal mitotic control and oncogenesis.. Journal of Theoretical Biology.

[49] Cone CD, Tongier M (1971). Control of somatic cell mitosis by simulated changes in the transmembrane potential level.. Oncology.

[50] Wonderlin WF, Strobl JS (1996). Potassium channels, proliferation and G1 progression.. Journal of Membrane Biology.

[51] Arcangeli A, Rosati B, Cherubini A, Crociani O, Fontana L (1997). HERG- and IRK-like inward rectifier currents are sequentially expressed during neuronal development of neural crest cells and their derivatives.. European Journal of Neuroscience.

[52] Arcangeli A, Rosati B, Crociani O, Cherubini A, Fontana L (1999). Modulation of HERG current and herg gene expression during retinoic acid treatment of human neuroblastoma cells: Potentiating effects of BDNF.. Journal of Neurobiology.

[53] Fischer-Lougheed J, Liu JH, Espinos E, Mordasini D, Bader CR (2001). Human myoblast fusion requires expression of functional inward rectifier Kir2.1 channels.. Journal of Cell Biology.

[54] Liu JH, Bijlenga P, Fischer-Lougheed J, Occhiodoro T, Kaelin A (1998). Role of an inward rectifier K+ current and of hyperpolarization in human myoblast fusion.. Journal of Physiology.

[55] Cone CD (1970). Variation of the transmembrane potential level as a basic mechanism of mitosis control.. Oncology.

[56] Olivotto M, Arcangeli A, Carla M, Wanke E (1996). Electric fields at the plasma membrane level: A neglected element in the mechanisms of cell signalling.. BioEssays.

[57] Altman GH, Horan RL, Martin I, Farhadi J, Stark PR (2002). Cell differentiation by mechanical stress.. The FASEB journal : official publication of the Federation of American Societies for Experimental Biology.

[58] Bustin SA, Benes V, Nolan T, Pfaffl MW (2005). Quantitative real-time RT-PCR-A perspective.. Journal of Molecular Endocrinology.

[59] Giulietti A, Overbergh L, Valckx D, Decallonne B, Bouillon R (2001). An overview of real-time quantitative PCR: Applications to quantify cytokine gene expression.. Methods.

[60] Nolan T, Hands RE, Bustin SA (2006). Quantification of mRNA using real-time RT-PCR.. Nature Protocols.

[61] Mauney JR, Nguyen T, Gillen K, Kirker-Head C, Gimble JM (2007). Engineering adipose-like tissue in vitro and in vivo utilizing human bone marrow and adipose-derived mesenchymal stem cells with silk fibroin 3D scaffolds.. Biomaterials.

[62] Meinel L, Fajardo R, Hofmann S, Langer R, Chen J (2005). Silk implants for the healing of critical size bone defects.. Bone.

[63] Meinel L, Hofmann S, Betz O, Fajardo R, Merkle HP (2006). Osteogenesis by human mesenchymal stem cells cultured on silk biomaterials: Comparison of adenovirus mediated gene transfer and protein delivery of BMP-2.. Biomaterials.

